# Risk factors of lower respiratory tract infection caused by *Stenotrophomonas maltophilia*: Systematic review and meta-analysis

**DOI:** 10.3389/fpubh.2022.1035812

**Published:** 2023-01-10

**Authors:** Yiwei Wang, Yizhi Wang, Hechen Rong, Zhonghong Guo, Jie Xu, Xiaoping Huang

**Affiliations:** ^1^Department of Infectious Diseases, The First Affiliated Hospital of Soochow University, Suzhou, Jiangsu, China; ^2^College of Medicine, Institute of Pharmaceutical Innovation, Wuhan University of Science and Technology, Wuhan, Hubei, China; ^3^Center for Clinical Laboratory, The First Affiliated Hospital of Soochow University, Suzhou, Jiangsu, China

**Keywords:** *Stenotrophomonas maltophilia*, respiratory tract infection, review, risk factors, meta-analysis

## Abstract

**Objective:**

To systematically evaluate the risk factors of lower respiratory tract infection caused by *Stenotrophomonas maltophilia* for better clinical treatment.

**Methods:**

PubMed, Embase, the Cochrane Library, Web of Science, China Journal full-text Database (CNKI), Wanfang Database (WanFang Data), VIP (VIP), and China Biomedical Literature Database (CBM) were selected and published by June 2022 about the risk factors of lower respiratory tract infection of *S. maltophilia*. Two researchers independently screened the literature, extracted data, and quality evaluation according to the inclusion and exclusion criteria. RevMan 5.4 software was used for meta-analysis.

**Results:**

A total of 18 articles were included, including 10 in English and 8 in Chinese. Meta analysis showed that the risk factors of lower respiratory tract infection caused by *S. maltophilia* included disease severity, hospitalization days, use of glucocorticoids, invasive procedures, use of broad-spectrum antibiotics and use of more than 3 Antibiotics. The OR values of patients with hospitalization, mechanical ventilation, use of more than 3 Antibiotics, endotracheal intubation and tracheotomy were the highest. Specific hospitalization days (OR = 14.56, 95% CI: 6.12~23.01), mechanical ventilation (OR = 14.16, 95% CI: 5.85~34.3), use of more than 3 Antibiotics (OR = 6.21, 95% CI: 1.24~31.14), tracheal intubation (OR = 6.07, 95% CI: 1.97~3.64), tracheotomy (OR = 3.77, 95% CI: 1.09~13.04).

**Conclusion:**

There are many risk factors for lower respiratory tract infection of *S. maltophilia*, which can occur in patients with severe illness, high APACHE-II score, invasive procedures, and the need for broad-spectrum antibiotics. In terms of the host, these patients are characterized by impaired immune function, severe illness and long-term hospitalization, which objectively leads to the infection of *S. maltophilia*. Therefore, strengthening the monitoring, prevention and control of patients with risk factors of *S. maltophilia* infection is conducive to reducing the risk of infection and death.

## Introduction

*Stenotrophomonas maltophilia* is an important conditional pathogen. It has become an important hospital-acquired pathogen for critically ill patients, and its ability to colonize respiratory epithelial cells and medical equipment makes it a ready-made colonizer for inpatients (1). *S. maltophilia* can cause bloodstream infection and pneumonia, and a few can cause skin, soft tissue, and urinary tract infections. It is one of the top ten HAP pathogens in ICU wards in European countries, accounting for 0.4–8.7% of HAP in all hospitals. Although the incidence of HAP caused by *S. maltophilia* is low, the mortality rate is about 50%, especially in immunocompromised patients with chronic respiratory diseases, cystic fibrosis, neutropenia, malignancy, and prolonged hospitalization, and can even be as high as 77% in immunocompromised patients (1). *S. maltophilia* is characterized by high resistance to antibiotics, including broad-spectrum β-lactam, aminoglycosides and carbapenem, which is associated with high morbidity and mortality in immunocompromised patients (2, 3). Important to mention here that previous therapy with broad-range antibiotics, especially carbapenem has been deemed as a risk factor for *S. maltophilia* infection (4, 5). The attributed mortality rates of *S. maltophilia* infections in pneumonia range between 25 and 75%, 26 and 28% in case of nosocomial bloodstream infections, and crude mortality rates of *S. maltophilia* bacteremia range from 21 to 69% (6, 7). In patients with cystic fibrosis (CF), multidrug resistant (MDR) bacteria can easily worsen the disease, limit the effectiveness of antibiotic treatment, and promote the progression of lung diseases. Fainardi et al. found that chronic infection by *S. maltophilia* was associated with increased risk of Pex and death/transplantation (8).The study on the risk factors of *S. maltophilia* lower respiratory tract infection is of great significance for studying the harm of *S. maltophilia* and taking prevention and control measures. At present, the articles at home and abroad on the risk factors of *S. maltophilia* infection have some shortcomings, such as small sample size, incomplete risk factor index, and so on (9). The purpose of this study is to evaluate the risk factors of lower respiratory tract infection of *S. maltophilia* by meta-analysis system, and to provide a theoretical basis for reducing the morbidity and mortality of maltophilia infection.

## Materials and methods

### Literature inclusion and exclusion criteria

Inclusion criteria are as follows: (1) Study types: case-control studies published at home and abroad that comprise two groups- SMA and non SMA. (2) Subjects: according to whether they were infected with *S. maltophilia*, the subjects were divided into two groups, and the diagnostic criteria of *S. maltophilia* pneumonia in this study were as follows: (1) new or progressive lung infiltration. (2) temperature >38°C or < 36.5°C, WBC count > 12 × 10^9^/L or < 4 × 10^9^/L, purulent endotracheal aspiration or sputum. (3) positive respiratory tract samples. (4) oxygenation decreased. Non-sma group: Patients without lower respiratory tract gram-negative bacilli infection in the same department were selected. (3) outcome index: the risk factors of *S. maltophilia* pneumonia, including age, disease, treatment, and other factors. It is expressed as odds ratio (OR), and its 95% confidence interval (CI) is calculated. (4) all documents have been published, and the publication period is up to June 2022.

Exclusion criteria: (1) missing data, no literature with odds ratio (OR) and 95% confidence interval (CI); (2) repeatedly published literature; (3) There were only abstracts, and the author had not yet obtained the full text of the literature; (4) case reports, reviews and animal experiments; (5) The quality of the literature was evaluated according to the Newcastle-Ottawa Scale (the Newcastle-Ottawa Scale, NOS) recommended by the Cochrane collaboration Network for use in non-randomized research groups, and the quality evaluation was a low-quality study; (6) the research methods are not similar.

### Literature search strategy

China National Knowledge Infrastructure Database (CNKI), Wanfang Database (WanFang Data), VIP (VIP), and China Biomedical Literature Database (CBM) were selected. (*S. maltophilia* OR *S. Maltophilia* OR SMA) AND (pulmonary infection OR lower respiratory tract infection OR pneumonia) AND (risk factors OR related factors OR influencing factors) was the keyword, and the references in the literature were further traced to expand the search scope. The search time range was from the establishment of the database to June 1, 2022. Select PubMed, Embase, the. Cochrane Library, Web of Science database, to [(*S. maltophilia*) OR (*S. Maltophilia*) OR (SMA)] AND [(lung infection) OR (lung infections) OR (pulmonary infection) OR (lower respiratory infection)] AND [(risk factors) OR (related factors) OR (influencing factors)] as the main search term. With PubMed as an example, the specific retrieval strategy was as follows:

#1[(*S. maltophilia*) OR (*S. maltophilia*) OR (SMA)]

#2 [(lung infection) OR (lung infections) OR (pulmonary infection) OR (lower respiratory infection)]

#3[(risk factors) OR (related factors) OR (influencing factors)]

#4 #1 AND #2 AND #3

### Literature screening and data extraction

According to the inclusion and exclusion criteria established in this study, two researchers strictly selected the literature and excluded the irrelevant literature. After a detailed reading of the included literature, the extracted literature information includes: (1) the basic information of the included study, including the first author, publication time, research type, etc; (2) the basic situation of the study subjects, including the number of cases, age, etc.; (3) research factors; (4) key elements of bias risk assessment; (5) outcome indicators and outcome measurement data concerned.

### Methodological quality evaluation

Newcastle-Ottawa Scale (NOS) was used to evaluate the quality of case-control studies. The scale consists of three parts: study population selection (4 items) comparability (1 item), and exposure assessment or outcome assessment (3 items), with a total of 8 items. NOS used the semi-quantitative principle to evaluate the literature quality, and the total score was 9.0–4 is classified as low-quality research, 5–6 as medium-quality research, and 7–9 as high-quality research. The included literature was independently evaluated by two researchers, and the evaluation results were cross-checked after the evaluation. In case of disagreement, the third researcher was consulted for arbitration.

### Data analysis

Revman 5.4 was used for statistical analysis. I^2^ was used to determine the heterogeneity of the included literature. When *P* > 0.1 and I^2^ < 50%, the fixed effects model was used. Otherwise, the random effects model is used. The OR and 95%CI were calculated for the enumeration data, and the weighted mean difference (WMD) and 95%CI were calculated for the measurement data. P ≤ 0.05 was considered statistically significant. Sensitivity analyses were performed by calculating OR and 95% confidence intervals for fixed-effect and random-effect models, and the results were compared between the two groups. Sensitivity analysis was performed by changing the data analysis model. The combined results are considered stable if no substantial changes occur after the model change (no contrary conclusion is reached after the model change). When the number of papers included in the individual risk factor analysis was ≥3, Begg's test was used to test publication bias.

## Results

### Literature search

**Figure 1:**
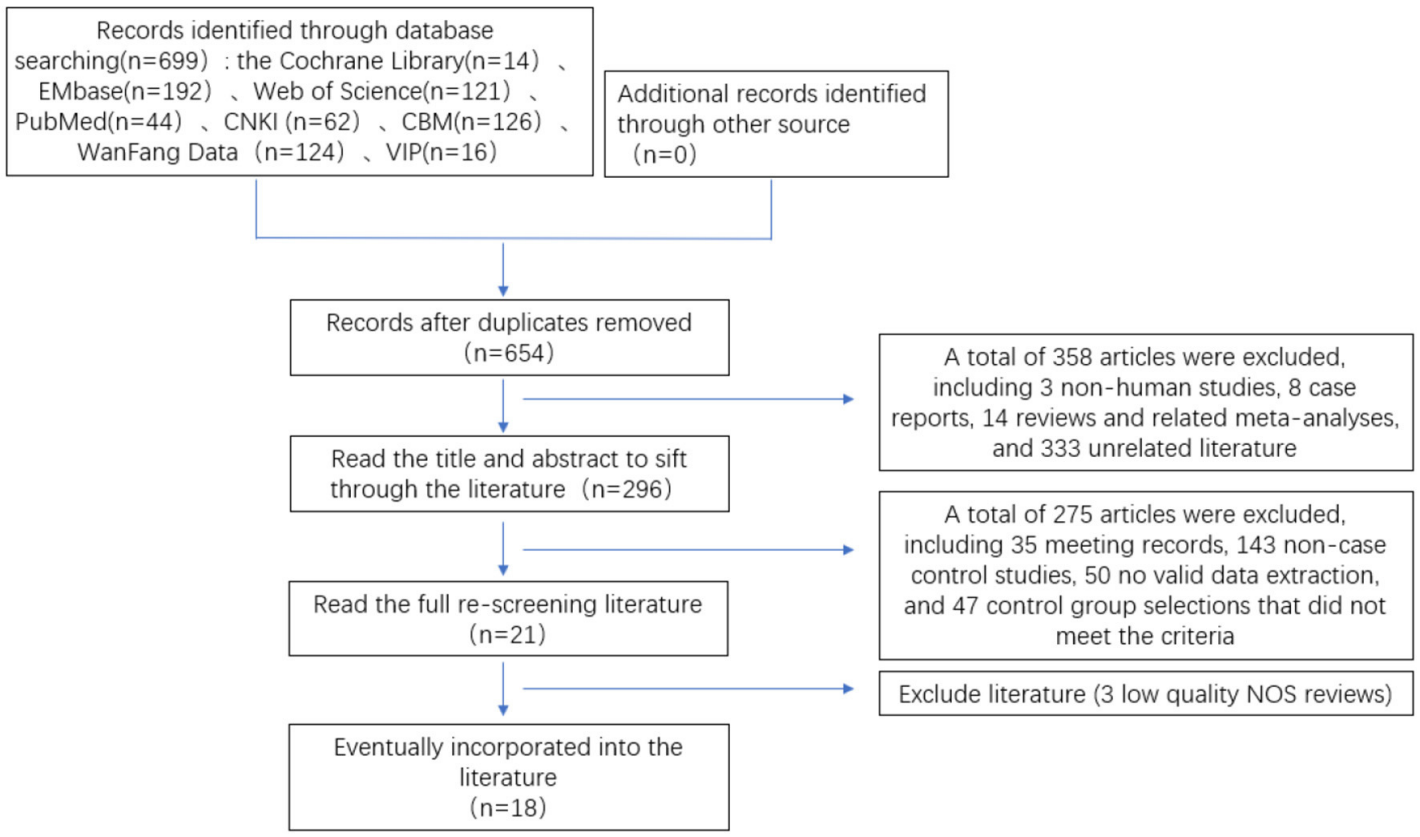
Flow chart of literature screening for meta-analysis of risk factors of lower respiratory tract infection caused by *S. maltophilia*.

First, 699 papers were preliminarily searched in the database through a search strategy, including 3 non-human experiments, 45 duplicates, 8 case reports, and 14 reviews and related meta-analyses. Preliminary screening of literature for inclusion by reading the title and abstract 375 papers were read, and 18 papers were finally included in the rescreening of the full text, including 10 in English and 8 in Chinese. There were 4,697 patients in total, 1,008 in the SMA group and 3,689 in the non-SMA group. The literature screening process and results are shown in Figure 1.

### Baseline characteristics of the studies

The 18 papers included in this study, published in 2001–2021, involved 4,697 patients, 1,008 in the SMA-infected group, 3,689 in the non-infected group, and 26 exposure factors for SMA infection. The NOS scale was used to evaluate the quality of 18 papers, including 17 high-quality papers and medium-quality papers. The basic features of the included literature are shown in Tables 1, 2.

**Table 1:** Include the basic characteristics of the literature.

**Study**	**Year**	**Design study**	**Area**	**Infection group**	**Non-infection group**	**Risk factors**
Raffenberg et al. (10)	2001	Case-control	Germany	16	95	06, 13, 14, 16, 17, 21
Dai et al. (11)	2006	Case-control	China	48	48	01, 02, 04, 13, 14, 16, 18, 19
Nseir et al. (12)	2006	Case-control	France	30	60	01, 02, 05, 06, 07, 10, 13, 15, 16, 17
Ansari et al. (13)	2007	Case-control	USA	54	108	01, 02, 04, 08, 09, 21, 23, 24, 25
Xu et al. (14)	2011	Case-control	China	35	140	01, 02, 03, 13, 14, 15, 18, 21, 23, 26
Mutlu et al. (15)	2011	Case-control	Turkey	23	45	02, 04, 05, 13, 17, 21, 23
Saugel et al. (16)	2012	Case-control	Germany	36	28	02, 06, 07, 08, 10, 15, 16, 17
Peng and Liu (17)	2013	Case-control	China	30	30	01, 02, 04, 21
Guo et al. (18)	2014	Case-control	China	42	84	01, 02, 03, 04, 06, 07, 10, 13, 14, 15, 18, 26
Lv and Zhang (19)	2014	Case-control	China	238	476	02, 03, 05, 06, 07, 08, 10, 12, 21, 22, 23, 24, 26
Hottal et al. (20)	2014	Case-control	Japan	54	167	02, 07, 08, 09, 10, 11, 12, 13, 16, 17, 18, 20, 21, 23, 25
Cha (21)	2015	Case-control	China	60	120	01, 02, 06, 07, 08, 10, 11, 12, 13, 16, 17, 20, 21, 22, 23, 24, 25
Cabaret et al. (22)	2016	Case-control	France	20	237	01, 02, 04, 06, 08, 09, 12, 14, 16, 19
Proceedings of reanimation (23)	2018	Case-control	France	93	380	04, 09, 10, 12, 16, 19, 21, 25
Liu et al. (24)	2018	Case-control	China	90	30	01, 02, 04, 05, 07, 08, 10, 13, 15, 16, 20, 21, 26
Shi et al. (25)	2019	Case-control	China	29	58	01, 02, 04, 05, 07, 08, 09, 10, 12, 13, 16, 17, 19, 21, 23, 24, 25
Ibn Saied et al. (26)	2019	Case-control	France	102	1,492	02, 04, 10, 11, 12
Imoto et al. (27)	2021	Case-control	Japan	8	91	02, 04, 08, 16, 18, 20, 22

01, Age, years; 02, Gender; 03, APACHE-II score >20; 04, Glucocorticoid; 05, Hospitalization days; 06, COPD; 07, Diabetes; 08, Solid malignant tumors; 09, Malignant tumors of the hematologic system; 10, Cardiovascular disease; 11, Kidney dysfunction; 12, Immunosuppression; 13, Mechanical ventilation; 14, Tracheal intubation; 15, Tracheotomy; 16, Central venous catheterization; 17, Urinary catheter; 18, Nasogastric tube; 19, Parenteral nutrition; 20, Operation; 21, Carbapenems; 22, β-lactamase inhibitor; 23, Aminoglycosides; 24, Quinolones; 25, Glycopeptides; 26, Use more than 3 kinds of antibacterial drugs.

**Table 2:** The quality of the included study was evaluated according to the Newcastle-Ottawa scale (NOS).

**Study ID/ NOS items**	**Is the case definition adequate?**	**Representativeness of the cases**	**Selection of controls**	**Definition of controls**	**Compatibility**	**Ascertainment of exposure**	**Same method of ascertainment for cases and control**	**Non-response rate**	**Total score**
Raffenberg et al.	*	*		*	*	*	*		6
Dai et al.	*	*		*	*	*	*		6
Nseir et al.	*	*		*	*	*		*	7
Ansari et al.	*	*	*	*	*	*	*		7
Xu et al.		*	*	*	*	*	*	*	7
Mutlu et al.	*	*		*	*	*	*		6
Saugel B et al.	*	*	*	*	*	*	*		7
Peng et al.	*	*		*	*	*	*	*	7
Guo et al.	*	*		*	*	*	*		6
Lv et al.	*	*		*	*		*		5
Hottal et al.	*	*		*	*	*	*	*	7
Cha et al.	*	*	*	*	*	*	*		7
Cabaret et al.	*	*		*	*	*	*		6
Sybille et al.	*	*			*	*	*	*	7
Liu et al.	*	*		*	*	*	*	*	7
Shi et al.	*		*	*	*	*	*	*	7
Ibn Saied et al.	*	*	*	*	*	*	*	*	8
Imoto et al.	*	*		*	*	*	*	*	7

^*^Representative studies meet this criteria.

The 18 papers included in this study were published between 2001 and 2021, involving 4,697 patients, 1,008 in the SMA infection group, 3,689 in the non-infection group, and 26 exposure factors to SMA infection. The NOS scale was used to evaluate the quality of 18 papers, including 12 high-quality papers and 6 medium-quality papers. The basic characteristics of the included literature are shown in Tables 1, 2.

### Meta-analysis of exposure factors for *S. maltophilia* pneumonia

Heterogeneity was tested for exposure factors such as Age, Sex, Diabetes, Solid tumors, Hematological malignancies, Cardiovascular disease, Renal insufficiency, Immunodeficiency disease, Endotracheal intubation, Surgery, and Use of β-lactamase inhibitors. Heterogeneity was acceptable (*P* > 0.10, I2 < 50%), and effect sizes were combined using fixed-effects models. APACHE-II scores >20, Hospitalization days, Glucocorticoid use, Parenteral nutrition, Use of more than 3 antibiotics, COPD, Tracheotomy, Mechanical ventilation, Indwelling Nasogastric tube, Central venous catheter, Urinary catheter, Carbapenems, Quinolones, Glycopeptides and Aminoglycosides (*P* < 0.10, I2 > 50%). The effect size was combined by a random effect model.

The meta-analysis showed that the risk factors of lower respiratory tract infection caused by *S. maltophilia* included APACHE-II score>20 (OR = 2.68, 95% CI:1.97~3.64), Hospitalization days (OR = 14.56, 95% CI: 6.12~23.01), Mechanical ventilation (OR = 14.16, 95% CI: 5.85~34.3), Tracheal intubation (OR = 6.07, 95% CI: 3.63~10.16) and Tracheotomy (OR = 3.77, 95% CI: 1.09~13.04), Indwelling nasogastric tube (OR = 3.00, 95% CI: 1.60~5.63), Central venous catheterization (OR = 2.16, 95% CI: 1.07~4.36), Glucocorticoid (OR = 2.08, 95% CI: 1.32~3.27), Carbapenems (OR = 3.69, 95% CI: 2.31~5.89), Aminoglycosides (OR = 2.57. 95% CI: 1.42~4.65), the Use of β-lactamase Inhibitors (OR = 1.76, 95% CI: 1.30~2.39), the Use of Glycopeptide Antibiotics (OR = 3.22, 95% CI: 1.48~7.01), and the Use of more than 3 Antibiotics (OR = 6.21, 95% CI:1.24~31.14). Lower respiratory tract infection of *S. maltophilia* was not significantly associated with Age, Sex, Parenteral nutrition, Use of Quinolone Antibiotics, Surgery, Urinary catheter, COPD, Diabetes, Cardiovascular disease, Solid tumor, Hematological malignant tumor, Immune deficiency disease, Renal insufficiency, and Immunodeficiency.

### Sensitivity analysis and publication bias

Sensitivity analysis showed that except for COPD and Urinary catheter, the meta-analysis results of all the results were stable (Table 3). Berger test was used to test the publication bias, and the results showed that *p* > 0.05, indicating that the publication bias included in the paper was not significant.

**Table 3:** Meta-analysis of risk factors of lower respiratory tract infection caused by *S. maltophilia*.

**Exposure factors**	**Included studies**	**Heterogeneity**	***p***	**Fixed-effect model (FEM)**	***p***	**Random-effect model (REM)**	***p***
**General condition**							
Age, years	9	0	0.99	0.04 (−0.10~0.17)	0.59	0.04 (−0.10~0.17)	0.59
Gender	16	0	0.81	0.99 (0.82~1.19)	0.93	0.99 (0.82~1.19)	0.93
APACHE-II score >20	3	93	<0.001	2.68 (1.97~3.64)	<0.001	5.56 (1.16~26.53)	0.03
Glucocorticoid	11	60	0.006	1.75 (1.37~2.23)	<0.001	2.08 (1.32~3.27)	0.002
Hospitalization days	6	92	<0.001	7.36 (6.11~8.62)	<0.001	14.56 (6.12~23.03)	<0.001
**Comorbid disease conditions**							
COPD	7	86	<0.001	1.45 (1.10~1.90)	0.008	1.89 (0.79~4.51)	0.15
Diabetes	7	34	0.17	0.96 (0.80~1.16)	0.67	1.00 (0.78~1.27)	0.97
Solid malignant tumors	9	0	0.81	1.29 (0.98~1.70)	0.07	1.27 (0.96~1.68)	0.09
Malignant tumors of the Hematologic system	5	42	0.14	0.90 (0.61~1.31)	0.57	0.88 (0.51~1.52)	0.65
Cardiovascular disease	9	37	0.12	0.87 (0.67~1.12)	0.26	0.95 (0.67~1.34)	0.77
Kidney dysfunction	3	0	0.88	0.85 (0.52~1.40)	0.52	0.85 (0.52~1.40)	0.53
Immunosuppression	7	40	0.13	1.07 (0.83~1.39)	0.59	1.06 (0.73~1.54)	0.74
**Invasive manipulation**							
Mechanical ventilation	10	80	<0.001	8.42 (6.06~11.71)	<0.001	14.16 (5.85~34.30)	<0.001
Tracheal intubation	4	0	0.89	6.07 (3.63~10.16)	<0.001	5.88 (3.54~9.75)	<0.001
Tracheotomy	6	84	<0.001	3.84 (2.47~5.95)	<0.001	3.77 (1.09~13.04)	0.04
Central venous Catheterization	11	80	<0.001	1,76 (1.35~2.30)	<0.001	2.16 (1.07~4.36)	0.03
Urinary catheter	6	81	<0.001	1.85 (1.26~2.72)	0.002	2.30 (0.81~6.52)	0.12
Nasogastric tube	5	60	0.04	3.17 (2.20~4.57)	<0.001	3.00 (1.60~5.63)	<0.001
Operation	4	32	0.22	1.32 (0.86~2.03)	0.20	1.31 (0.70~2.44)	0.40
**History of antibacterial Drug use**							
Carbapenems	12	77	<0.001	2.91 (2.38~3.55)	<0.001	3.69 (2.31~5.89)	<0.001
β-lactamase inhibitors	3	10	0.33	1.76 (1.30~2.39)	<0.001	1.73 (1.18~2.55)	0.005
Aminoglycosides	7	50	0.06	2.13 (1.50~3.03)	<0.001	2.57 (1.42~4.65)	0.002
Quinolones	4	89	<0.001	0.82 (0.62~1.08)	0.16	1.41 (0.50~3.93)	0.52
Glycopeptides	5	76	0.002	2.72 (1.97~3.76)	<0.001	3.22 (1.48~7.01)	0.003
Use more than 3 kinds of antibacterial drugs	4	93	<0.001	2,47 (1.81~3.37)	<0.001	6.21 (1.24~31.13)	0.03

## Discussion

*S. maltophilia* belongs to non-fermented, non-spore aerobic Gram-negative bacilli. It is a common conditional pathogen, widely distributed in nature, and sojourns in sewage, soil, animals and human bodies. It also exists in hospital environments, such as hospital air, bed sheets, medical instruments, and various tubing devices (dialysis devices, artificial respirators, ventilation pipes, and oxygen humidification tanks). In recent years, with the wide application of immunosuppressants, broad-spectrum antimicrobials, and glucocorticoids in clinical treatment and the development of various invasive medical operations, the infection rate of *S. maltophilia* is increasing year by year. *S. maltophilia* has become one of the most important pathogens of nosocomial infection (26). *S. maltophilia*, reported to be found in approximately 3.7% of discharged patients, is the third most common non-fermentative Gram-negative bacilli causing hospital-acquired infections. Pseudomonas aeruginosa and Acinetobacter are the first and second most common bacteria causing human health-related infections, respectively. Surveillance of drug resistance of *S. maltophilia* in China CHINET data in 2021 showed that the isolation rate of *S. maltophilia* accounted for 3.9% of Gram-negative bacteria, ranking fifth (28). Data in the past 16 years show that the number of isolated strains of *S. maltophilia* increased year by year, from 877 strains in 2005 to 2,156 strains in 2012 and 6,465 strains in 2018, accounting for about 10.9% of non-fermentative Gram-negative bacilli (29). Most of the pathogenic strains of *S. maltophilia* have obvious multiple drug resistance, even pan or total drug resistance. Because the patients with lower respiratory tract infection caused by *S. maltophilia* are seriously ill, and most of the strains are drug-resistant, it is difficult to treat, which often leads to an increase in medical expenses, prolonged hospitalization and higher mortality. Therefore, early targeted treatment is the key to reducing the death rate of *S. maltophilia* infection. This study is based on the general condition of the patients, underlying diseases, invasive procedures, and antibiotics. Four aspects of the situation were used to evaluate the exposure factors of lower respiratory tract infection caused by *S. maltophilia* based on evidence-based medicine to screen out the risk factors of *S. maltophilia* infection.

### General and underlying diseases associated with lower respiratory infections caused by *S. maltophilia*

**Figure 2:**
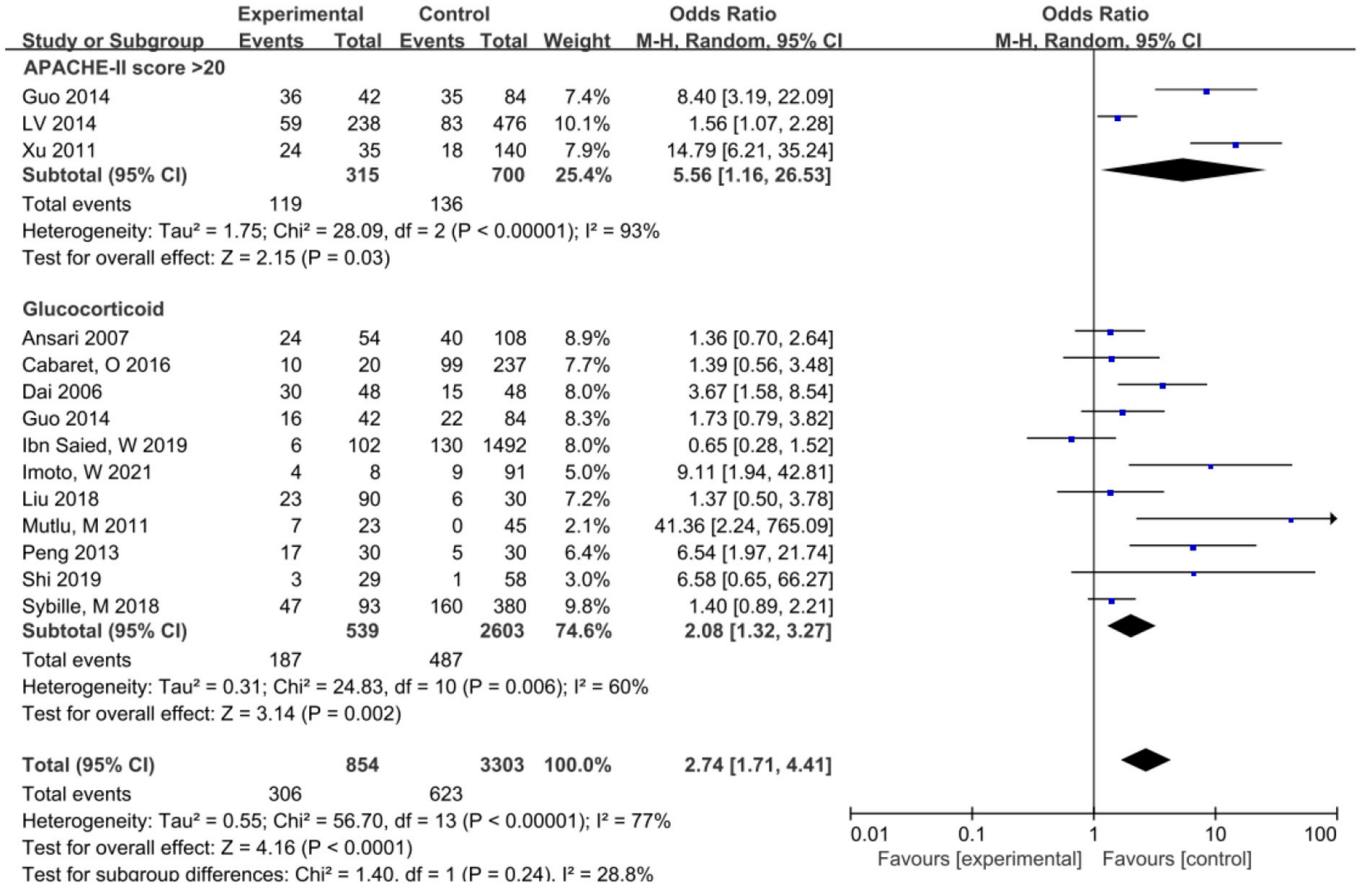
Meta-analysis of the risk factors of lower respiratory tract infection caused by *S. maltophilia* in patients with general conditions and underlying diseases.

*S. maltophilia* pneumonia was associated with general conditions including APACHE-II score >20, Hospitalization days, and use of Glucocorticoids. APACHE-II score is the most widely used critical condition assessment tool at home and abroad, which is used to assess the severity of the disease, prognosis, and risk of death. The study reported that the independent risk factor associated with the mortality rate of *S. maltophilia* infection was APACHE score >20 (30). In this study, the APACHE-II score of patients with SMA infection >20 was higher than that of patients without *S. maltophilia* infection, and the OR value was 2.68, indicating that *S. maltophilia* pneumonia was closely related to the severity of the disease. Glucocorticoids play an immunosuppressive role and induce cellular immunodeficiency, thereby increasing the host's susceptibility to various viruses, bacteria, fungal, and parasites (31). Long-term and heavy use of Glucocorticoids is a high-risk factor for multiple drug-resistant bacteria and fungal infections (32). The analysis of this study showed that Glucocorticoid use was closely related to lower respiratory tract infection of *S. maltophilia* (OR = 2.08), indicating that Glucocorticoid increased the incidence of *S. maltophilia* pneumonia. Hospitalization days is also an important factor in the development of *S. maltophilia* pneumonia. For *S. maltophilia* pneumonia, age ≥ 65 years, hospital stay ≥ 28 days and inappropriate antibiotic treatment were identified as risk factors for 30-day mortality in cancer patients. A study in Taiwan investigated 406 patients with *S. maltophilia* pneumonia and found that before the onset of *S. maltophilia* pneumonia, about 60% of the patients were hospitalized for more than 28 days (33). Related studies have reported that patients with multiple underlying diseases are risk factors for developing *S. maltophilia* pneumonia (34). However, by combining various underlying diseases between the experimental group and the control group, we did not find any statistical difference between the two groups, and did not find that suffering from underlying diseases can increase the incidence of *S. maltophilia* pneumonia, which may be biased due to differences in disease severity and drug treatment efficacy (Figure 2).

### Invasive manipulation and lower respiratory infections caused by *S. maltophilia*

**Figure 3:**
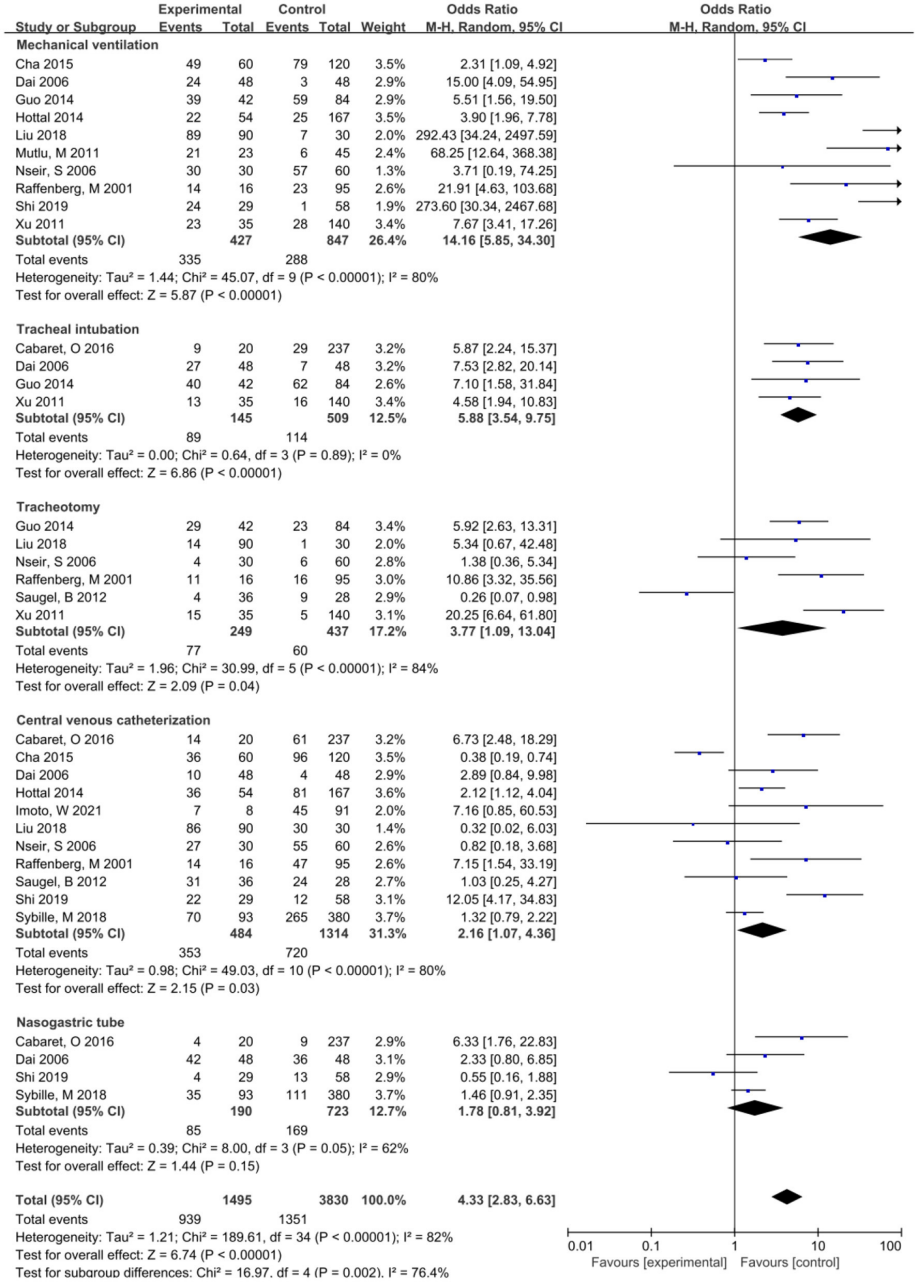
Meta-analysis of the effect of invasive procedures on risk factors of lower respiratory tract infection caused by *S. maltophilia*.

This study showed that the risk factors associated with invasive operation included mechanical ventilation, tracheal intubation, tracheotomy, central venous catheterization, and indwelling nasogastric tube, among which mechanical ventilation and *S. maltophilia* pneumonia had the highest OR (OR = 14.16), followed by tracheal intubation (OR = 6.07) and tracheotomy (OR = 3.77). Indwelling nasogastric tube (with OR = 3.00) and central venous catheterization (with OR = 2.16).This study suggests that invasive procedures such as mechanical ventilation, endotracheal intubation, and tracheotomy are high risk-factors for lower respiratory tract infection of *S. maltophilia* (Figure 3). *S. maltophilia* can colonize epithelial cells on the surface of the respiratory tract and medical equipment (35). Invasive operations such as mechanical ventilation, tracheal intubation, or tracheotomy can destroy the basic defense barrier of the human body, destroying respiratory mucosal barrier and the decrease or disappearance of airway self-purification ability (12, 33). The longer the invasive operation, the more severe the airway damage, and the higher the risk of lower respiratory tract infection *S. maltophilia* (36). Central venous catheterization and indwelling nasal catheters increase the risk of lower respiratory tract infection caused by *S. maltophilia*, which is consistent with the findings of Minako Mori (37). Central venous catheterization and indwelling gastrointestinal tubes can also destroy the body's defense barrier. *S. maltophilia* is easy to invade through the catheterization site and adhere to the inner surface of the catheter to grow and form a bacterial biofilm. Most of the patients with catheterization are in critical condition, weak physique, poor nutrition, low autoimmune function, often complicated with serious underlying diseases, and are prone to drug-resistant *S. maltophilia* infection (12, 38). In addition, there are a variety of drug-resistant mechanisms, resulting in repeated infections of the body. And it has been reported that 60% of conditional bacterial infections are related to their biofilms (39). Therefore, it is necessary to strictly grasp the indications of invasive surgery and shorten the time of invasive surgery as soon as possible in order to reduce the incidence of drug-resistant *S. maltophilia* infection.

### Antibacterial drug use and lower respiratory infections caused by *S. maltophilia*

**Figure 4:**
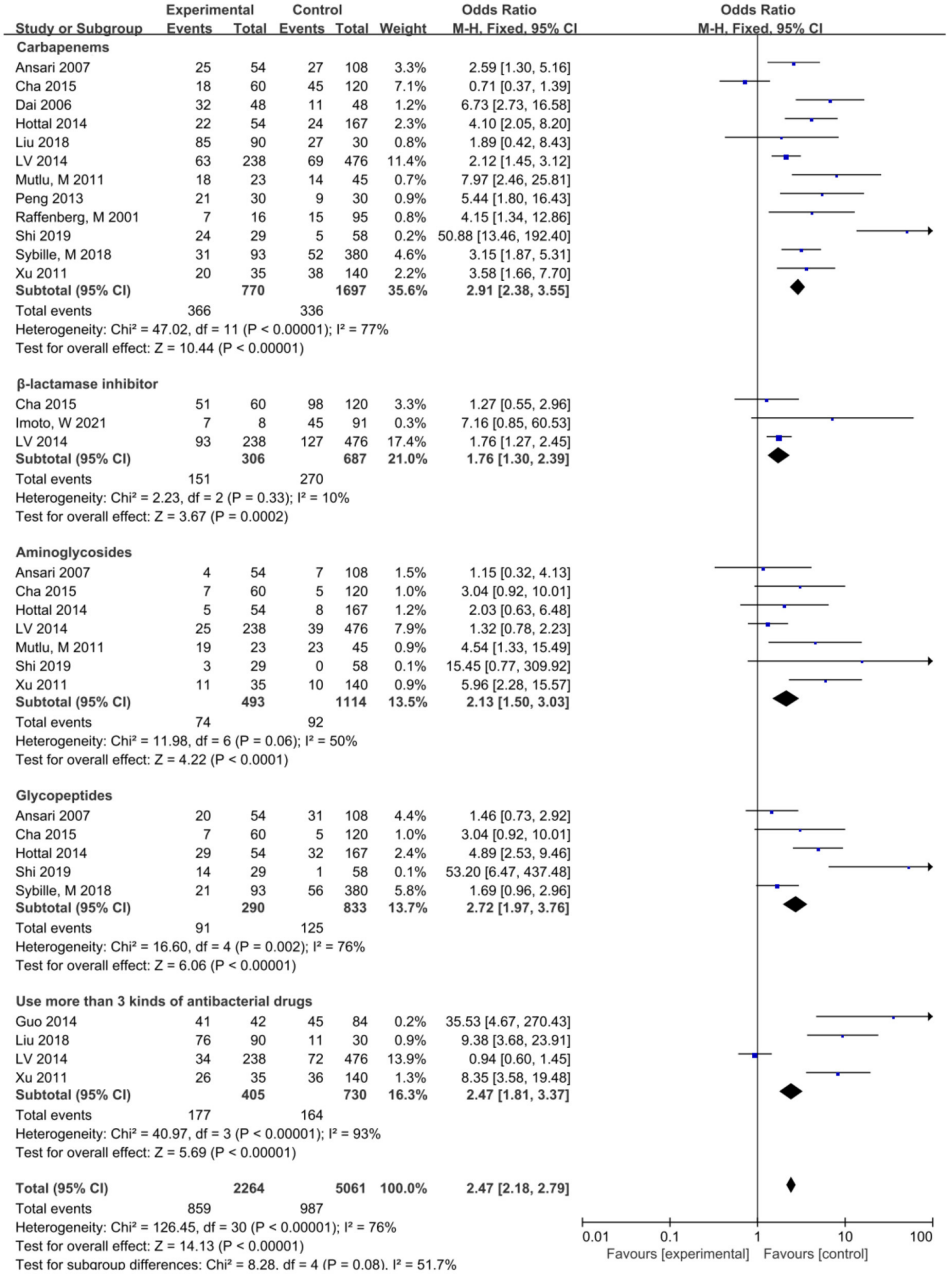
Meta-analysis of the effect of antibiotics on the risk factors of lower respiratory tract infection caused by *S. maltophilia*.

This study showed that the risk factors associated with antimicrobial use included carbapenems before *S. maltophilia* isolation, β-lactamase inhibitors, aminoglycosides, glycopeptides, and combination of antimicrobials (Figure 4). *S. maltophilia* showed a high level of inherent resistance to a variety of structurally unrelated antibiotics, including β-lactams, quinolones, aminoglycosides, tetracyclines, disinfectants, and heavy metals (40). The main determinants of internal drug resistance of *S. maltophilia* are the low permeability of multidrug efflux pump and outer membrane (40, 41). The reason for the high resistance of *S. maltophil* to β-lactamases is that *S. maltophil* has two inducible β-lactamases encoded by chromosomes, β-lactamase L1 (belonging to an Ambler class B zinc-dependent metalloenzyme) and β-lactamase L2 (belonging to an Ambler class A serine active site β-lactamases).When *S. maltophilia* is exposed to β-lactam antibiotics, L1 and L2βlactamases protect bacterial cells by hydrolyzing β-lactam (42, 43). The resistance of *S. maltophilia* to quinolones is mainly caused by mutations in the gyrA and parC genes at the target site (QRDRs) of DNA rotase, which is also related to outer membrane barrier and high-efficiency efflux pump (20, 44). The resistance of *S. maltophilia* to aminoglycoside antibiotics can be attributed to the temperature-dependent resistance caused by aminoglycoside modifying enzymes, EFFLUX pumps, and outer membrane proteins (45, 46). The distribution of aminoglycoside modifying enzyme in *S. maltophilia* was not uniform. According to a recent analysis of the genomes of 1,305 *S. maltophilia*, aminoglycoside phosphotransferases (APH) are encoded in 66% of strains and distributed in many gene groups, such as Sm6 and Sm5; aminoglycoside phosphotransferases (APH) are inherently resistant to aminoglycosides. Aminoglycoside acetyltransferase (AACs) is encoded in 6.1% of the strains, mainly belonging to genotypes Sgn4, Sm4b and Sm15, which can reduce sensitivity to aminoglycosides (4, 47). Selection of antimicrobial agents given the high level of inherent drug resistance and increasing drug resistance rate of *S. maltophilia* infection, it is a challenge to choose an appropriate antibacterial regimen to treat *S. maltophilia* infection. This study showed that carbapenem was associated with lower respiratory tract infection in SMA with the largest OR (OR = 3.69), followed by glycopeptide drugs (OR = 3.22), aminoglycoside drugs (OR = 2.57) and β-lactamase inhibitors (OR = 1.76). It is suggested that when clinical use of this type of drug is not effective, attention should be paid to the possibility of SMA lower respiratory tract infection. More kinds of antibiotics, longer time, and more times of replacement can significantly increase the risk of SMA lower respiratory tract infection. There is an equivalent cascade relationship between the types of antibiotics used and SMA infection, and more than 3 kinds of antibiotics are independent risk factors for lower respiratory tract infection of *S. maltophilia* (20), which is similar to the results of the study.

TMP-SMX is considered to be a first-line drug for the treatment of *S. maltophilia* infection. However, adverse reactions such as nephrotoxicity, allergic reactions and drug resistance may limit their use. These characteristics make infection caused by *S. maltophilia* a particular challenge and additional treatment options are urgently needed. The approach to *S. maltophilia* infections with combination aztreonam and avibactam therapy continues to gain interest due to the potential capacity for evasion of both chromosomally encoded L1 and L2 β-lactamases (48, 49). Mojica et al. found that the novel ceftazidime-avibatam and amtrinam (CZA-ATM) had excellent sensitivity rates, especially as it demonstrated that strains recovered from blood and other infections (mostly respiratory samples) were more sensitive to CZA-ATM than TMP-SMX. The activity of CZA-ATM was comparable to that of TMP-SMX. These results therefore confirm previous findings regarding the effectiveness of CZA-ATM against *S. maltophilia in vivo* and *in vitro*. Further observational and controlled studies are needed to increase clinical data on the efficacy and safety of CZA-ATM therapy (50). Cefdinol is the first iron carrier coupled cephalosporin approved for the treatment of human bacterial infections. Its stability to serine and metallo-β-lactamases has aroused great interest in the treatment of multi-drug resistant Gram-negative infections. Karlowsky et al. reported that continuous annual SIDERO-WT surveillance studies (2014–2019) found that *S. maltophilia* (98.6%) was highly sensitive to cefdilol (98.6%) *in vitro* sensitivity data for cefopiol and its controls from Gram-negative clinical isolates from North America and Europe (51). It must be emphasized, however, that Clinical and Laboratory Standards Institute (CLSI) does not provide MIC breakpoints for the explanation of cefdilol in *S. maltophilia*, which plays a major role in MDR and extensively drug resistant (XDR) infections (52). The mechanism of drug resistance of *S. maltophilia* is not clear.

### Limitations of this study

The main results are as follows: (1) The time span of the literature published in this study is large, and the quality of the literature is uneven, which leads to a certain heterogeneity in the meta-analysis of risk factors. (2) The sample size included in the literature in this study is quite different, which may lead to some heterogeneity in the combined analysis of some factors. (3) The intensity of perception and control and measures are not in different countries and regions. The same makes some of the research factors have a certain heterogeneity. (4) Only the published literature is collected, but no unpublished literature is obtained, so there may be some publication bias. Therefore, it is necessary to include a larger sample for analysis in future research to provide more strong evidence support.

## Conclusion

Most *S. maltophilia* strains are drug-resistant, which often leads to increased medical costs, prolonged hospitalization and increased mortality. From the host point of view, most of these patients are characterized by low immune function, serious illness and need to be hospitalized for a long time. The results of this study showed that the disease severity, hospitalization days, use of glucocorticoids, invasive procedures, broad-spectrum antibiotics and multiple antibiotics were the risk factors of *S. maltophilia* lower respiratory tract infection. This is the result of the combined action of host and medical factors. Therefore, early targeted treatment is the key to reduce the mortality of Streptococcus maltophilia infection.

## Data availability statement

The original contributions presented in the study are included in the article/supplementary material, further inquiries can be directed to the corresponding authors.

## Author contributions

YiwW and YizW collected information, analyzed data used in the systematic review and meta-analysis, and drafted the work. HR and ZG edited the subsequent versions of the manuscript. All authors contributed to the article and approved the submitted version.
